# Metabolomic profiling in treated mucopolysaccharidosis IH reveals candidate biomarkers and adjunctive therapeutic pathways

**DOI:** 10.64898/2026.09.04.26362233

**Published:** 2026-09-07

**Authors:** Troy C. Lund, Ryan H. Peretz, Patricia I. Dickson, Jennifer K. Yee, Michelina Iacovino, Kent D. Taylor, David Elashoff, Ellen Fung, Bradley S. Miller, Paul J. Orchard, Lynda E. Polgreen

## Abstract

Both severe (Hurler syndrome; MPS IH) and attenuated (Hurler-Scheie or Scheie syndrome; MPS IA) forms of mucopolysaccharidosis I (MPS I) arise from the same underlying enzyme deficiency; however, they differ in the onset, persistence, and severity of key clinical features. These include skeletal abnormalities, joint contractures, cardiac disease, and neurocognitive and neurobehavioral impairment, which is not fully alleviated with either enzyme replacement (ERT) or hematopoietic cell transplantation (HCT). The objective of this study was to identify metabolic differences between MPS IH and MPS IA that could result in meaningful biomarkers and targeted adjunctive therapies to address unmet clinical needs in MPS IH treated with HCT. We performed plasma metabolomics in patients with MPS IH treated with HCT (N=17) or MPS IA treated with ERT (N=8). Welch’s two-sample t-test was used to identify metabolites that differed significantly between groups. Statistical significance was evaluated based on p<0.05. After controlling for multiple testing, we used a false discovery rate of q<0.05. We identified 125 compounds that were significantly different between MPS IH and MPS IA. Of those metabolites, 14 had a q<0.05. Individuals with MPS IH had increased metabolites in the sphingolipid, beta-oxidation, amino acid catabolism, and glycosaminoglycan pathways compared to MPS IA. Persistent metabolic differences were observed in MPS IH treated with HCT compared to MPS IA treated with ERT, pointing towards additional biological processes that may contribute to disease progression in MPS IH after HCT. Although these results require further confirmation, they provide a foundation to guide future investigations towards potential biomarkers or targeted adjunctive therapies.

## Full Text Availability

The license terms selected by the author(s) for this preprint version do not permit archiving in PMC. The full text is available from the preprint server.

